# Use of long-lasting insecticide-treated bed nets in a population with universal coverage following a mass distribution campaign in Uganda

**DOI:** 10.1186/s12936-016-1360-0

**Published:** 2016-06-07

**Authors:** Humphrey Wanzira, Henry Katamba, Denis Rubahika

**Affiliations:** National Malaria Control Program, Ministry of Health, Kampala, Uganda

**Keywords:** Long-lasting insecticide-treated bed nets, Universal coverage, Malaria

## Abstract

**Background:**

Uganda conducted an LLIN mass distribution campaign in 2013 with the goal of achieving universal coverage. Using data from the 2014 malaria indicator survey, this analysis estimated the proportion of the population with access to an LLIN that slept under one the night before the survey and factors associated with not using an LLIN in households that had achieved universal coverage.

**Methods:**

This was a secondary data analysis using the 2014 malaria indicator survey dataset. The outcome was use of an LLIN among households that achieved universal coverage, while independent variables include age, gender, number of household members, residence, number of sleeping rooms, spraying of rooms with insecticide, number of children under 5 years of age, number of women of child-bearing age, relationship structure and community distribution of ant-malarial medicine.

**Results:**

Overall, 3361 (62 %) households of the 5345 achieved universal coverage and were included in the analysis giving a total population of 14,450 individuals. Of these, 11,884 (80.10 %) reported to have slept under an LLIN the night before the survey. Children between 6 and 14 years were significantly less likely to use an LLIN when compared to those under 5 years (75.26 vs 83.12 %), [adjusted OR, 1.29 (1.11–1.49), p = 0.001]. The odds of not using an LLIN, significantly increased from households with five members when compared to those that had one member (79.53 vs 84.88 %), [adjusted OR, 2.16 (1.38–3.38), p = 0.001] and rising even further in households with six or more members (78.04 vs 84.88 %), [OR, 2.27 (1.36–3.71), p = 0.003].

**Conclusions:**

This analysis has showed that 80 % of the population used an LLIN among households that achieved universal coverage following the 2013 mass distribution campaign, especially among children under 5 years, an operational success in this category. However, children between 6 and 14 years and individuals from households with five or more numbers are less likely to use the LLINs. In order to improve usage in these categories, it may require re-focusing the behaviour change communication message to be all-inclusive, especially in era of universal coverage, and to increase the number of LLINs distributed in households with more than four members during future mass distribution campaigns, respectively.

## Background

Increasing the coverage and use of long-lasting insecticide-treated bed nets (LLIN) is the most promoted malaria vector control prevention strategy in malaria endemic countries, in line with the World Health Organization (WHO) recommendations [1, 2]. LLINs prevent malaria by serving as physical barriers between mosquito vectors and individual users and the impregnated pyrethroid insecticide is repellent and toxic to mosquitoes [3–5].

In Uganda, malaria is endemic in over 90 % of the country’s regions and the National Malaria Control Programme (NMCP) estimates that between 30 and 50 % of outpatients’ visits, 15–20 % of hospital admissions and 20 % of hospital deaths are due to malaria with the biggest burden born by children under 5 years of age and pregnant women [6]. Therefore, the drive to scale up LLIN coverage in order to increase access and subsequently use is a significant goal for the NMCP as a malaria prevention strategy. Over the last 15 years, a number of approaches to improve coverage have been pursued, that included waving of taxes on imported nets in 2000 and finally in 2007, the adoption of the plan for LLIN universal coverage of the whole population, defined as one net for every two people [2, 6]. The first phase that targeted distribution of LLINs to children under 5 years and pregnant mothers in 2010, was the starting step in this approach. It was complemented by the continuous distribution of LLINs through the antenatal clinics (ANC) and expanded programme for immunization (EPI) services.

In 2013, Uganda conducted its first mass LLIN distribution campaign, where over 20 million nets were distributed free-of-charge to all registered households country wide, to over 41 million registered individuals, with the objective of achieving universal coverage. The rational of this strategy is that it would increase LLIN coverage and access, more so by closing equity related gaps that had been a source of differences in LLIN ownership [7–9].

However, merely owning a net or being able to access one does not automatically translate into its use, as previous studies have often reported [10–12]. Indeed, the 2014 Uganda malaria indicator survey (MIS) showed that 79 % of the household population had access to an LLIN (measured by the proportion of the population that could sleep under an LLIN if each LLIN in the household were used by up to two people) within their household and yet 69 % of these households slept under it [13]. A number of studies have been conducted to explore factors associated with not using an LLIN in different populations and circumstances of LLIN coverage [14–16], but there is limited information, specifically looking at LLIN use in a population that has achieved universal coverage.

Using data from the 2014 malaria indicator survey, the objectives of this analysis were to estimate the proportion of the population that had used an LLIN among households that had achieved universal coverage and factors associated with not using one. This is important in this era where malaria stakeholders are now advocating for universal LLIN coverage [1, 2, 17], meaning that understanding the drivers of use, in this group, is critical especially in re-focusing of malaria related behavioural change communication messages.

## Methods

This is a secondary data analysis using the 2014 Uganda malaria indicator survey dataset following the LLIN mass distribution campaign of 2013.

### Description of the LLIN mass distribution campaign

The aim of the LLIN mass distribution campaign in Uganda was a drive towards achieving LLIN universal coverage, defined as one net for every two people [2]. The design of the campaign largely followed international protocols, as developed by Roll Back Malaria (RBM)/Alliance for Malaria Prevention [18]. The guidelines on mass campaign [19] were developed jointly by the Ministry of Health and stakeholders, including the President’s Malaria Initiative (PMI), The Department of International Development (DFID)/UK Aid, and the Global Fund for AIDS, Tuberculosis and Malaria (GFATM), the WHO, World Vision and UNICEF Country Offices. The guidelines had seven key elements namely: campaign management structure, financing and financial management of the LLIN campaign, procurement, transportation and storage of LLINs, household registration and LLIN allocation, distribution of LLINs to beneficiaries, training and supervision and advocacy, social mobilization and behaviour change communication. In brief, the implementation involved introducing the campaign activities at the district level, training and sensitisation of all key personnel and supervisors who were involved in the exercise, registration of all households and the number of households’ members in each household at the village level by the village health teams (VHTs). Distribution of LLINs took place at the village level, the lowest administrative unit, mainly through a fixed-point distribution approach, at an agreed place(s) that was/were easily accessible. However, a door-to-door methodology was an added approach in urban settings, in consideration of the low turn up at the fixed-point selected places. All registered households received one net for every two household members (with number of bed nets rounded up for those with odd numbers of household members), irrespective of the previous bed nets they had. To ease distribution, Uganda’s 112 districts were grouped into nine categories, a pilot phase followed by eight distribution waves. The distribution was conducted as follows; Pilot (4 districts): Sept 2012, Wave 1 (2 districts): May 2013, Wave 2 (16 districts): June 2013, Wave 3 (16 districts): Oct 2013, Wave 4 (18 districts): Nov 2013, Wave 5 (15 districts): Jan 2014, Wave 6 (17 districts): June 2014, Wave 7 (17 districts): June 2014 and Wave 8 (7 districts): August 2014. By the end of the campaign, more than 20 million LLINs were distributed to approximately 41 million individuals.

### Description of the 2014 malaria indicator survey (MIS)

The survey was conducted during the months of December 2014 and January 2015, approximately 3 months after the last wave of the LLIN mass distribution campaign. Households were selected using a stratified two-stage cluster design from 210 enumeration areas, representing all the regions of the country with 44 in urban areas and 166 in rural areas. An EA was defined as a natural village in rural areas and a city block in urban areas. In the first stage, 20 sampling strata (derived from 10 regional domains: Central 1 and 2, East Central, Kampala, Mid-Northern, Mid-Western, Mid-Eastern, South-Western and West Nile) were created and EAs were selected independently from each stratum by a probability-proportional-to-size selection. In the selected EAs, a complete listing of households and a mapping exercise was conducted in November 2014, with the resulting list of households serving as the sampling frame for the selection of households in the second stage. The average EA size was 94 households in urban areas and 77 households in rural areas, with an overall average size of 80 households per EA. In the second stage of the selection process, 28 households were selected in each EA by equal probability systematic sampling. A total of 5802 households were selected for the 2014 MIS, of which 5494 were occupied. Of the occupied households, 5345 were successfully interviewed, yielding a response rate of 97 percent. The response rate among households in rural areas was slightly higher (98 percent) than the response rate in urban areas (96 percent) [13]. The main reason for non-response was failure to find individuals at home despite up to four repeated visits to the household. Two questionnaires were used to collect survey data, the household questionnaire (administered to all household heads) and women’s questionnaire (administered to all women aged 15–45 years in a selected household). Informed consent was obtained from all participating heads of households and women of child-bearing age in the households that participated in the survey. Particular to LLIN coverage and use, the interviewers asked about type and source of LLINs (with a question focused on whether the bed net was obtained through the campaign and if so, the date it was received), interviewers also observed the bed nets in the households and asked whether individuals had slept under the net the night before the survey.

For the specifics of this study, information from two 2014 MIS datasets was used; the household and individual member datasets. The household dataset contains information on all residents in the selected households regarding the characteristics of each person who was listed to have spent the night before the survey in the household like age, gender and relationship to the head of the household, number of household members, residence, number of sleeping rooms in a household, spraying of rooms with insecticide, community distribution of malaria medicine, ownership and use of mosquito bed nets and wealth index. The individual member dataset has one record for every household member and includes additional variables like number of children under 5 years, number of women of child-bearing age and mother’s highest education attainment.

### Study variables

The main dependent variable of interest in this analysis was the use of an LLIN defined as the proportion of de-facto household population that slept under an LLIN the night before the survey among households that achieved universal coverage. Independent variables considered for LLIN use included; age, gender, number of household members, residence, number of sleeping rooms, spraying of rooms with insecticide, number of children under five years, number of women of child-bearing age (15–45 years), adult relationship structure in the household, wealth index (poorest, poor, medium, rich, richest), mothers highest education attainment and community distribution of anti-malarial medicine.

### Data analysis

Stata version 14 (Statcorp, College Station, Texas, USA) was used for all data analysis. For this analysis, the household and individual member datasets were merged to derive the outcome measure of LLIN use and factors associated. Due to the non-proportional allocation of the sample in the different regional domains at the second sampling stage (28 households were selected in each EA by equal probability systematic sampling), the sample was not self-weighting. Weighting factors were calculated based on the population of the selected regional domains and added to the MIS datasets so that any results with the regional weight factored into it would be representative at the national and regional level as well as the survey domain level. Details of how the weighting for the different regional domains was estimated are available in the 2014 MIS report [13]. Therefore, for this study, only weighted survey data is presented in this manuscript. The distributions of study participant baseline characteristics were presented as frequencies with respective proportions. A multivariate logistic regression model with a survey function was used to assess for the factors influencing the use of LLINs in the population to derive first the crude and then the adjusted odds ratio with its respective confidence interval. In all analyses, a p value of <0.05 was taken as statistically significant.

### Ethical considerations

The 2014 MIS was approved by two ethical review bodies that included the Makerere University School of Biomedical Sciences Higher Degrees Research and Ethics Committee (SBS-HDREC) and the Uganda National Council for Science and Technology (UNCST). All participants interviewed gave their informed consent to participate in the 2014 MIS in addition to granting permission that information from survey could be published. The data used in this analysis was anonymous with no individual names of participants were captured.

## Results

### Baseline participants’ characteristics

**Fig. 1:**
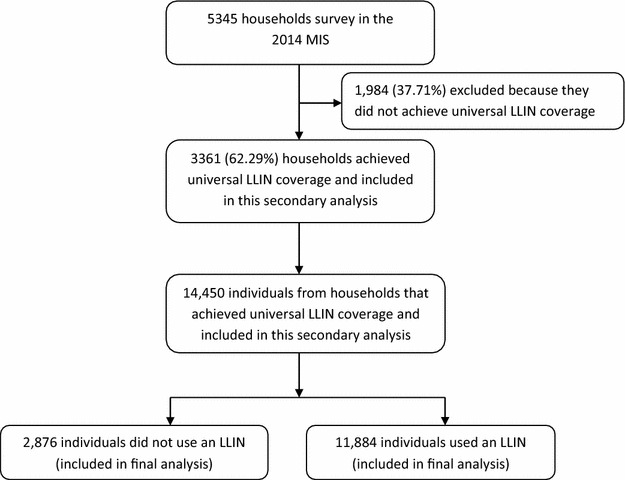
Study profile

In this analysis, a total of 14,450 individuals were included, from the 3361 households that had achieved universal LLIN coverage (Fig. 1). Over half of the participants (53.25 %) were 15 years and older, followed by those between 6 and 14 years (25.83 %) and the least percentage were those below 5 years of age (20.92 %) as shown in Table 1. Females (51.57 %) were slightly more than males, most participants were from a rural setting (80.41 %) and the south western region contributed the biggest percentage (16.38 %) of all the 10 regions while Kampala had the least (5.09 %).

**Table 1:** Baseline characteristics of survey participants

Characteristic	Distribution of participants	
	Total population N = 14,450	Percentage
*Age categories* (*years*)^*a*^		
0–5	3021	20.92
6–14	3731	25.83
15–45	5938	41.10
>45	1756	12.15
*Gender*		
Male	6999	48.43
Female	7451	51.57
*Region*		
Central 1	1505	10.42
Central 2	1236	8.55
East central	1094	7.57
Kampala	736	5.09
Mid-North	1729	11.97
Mid-Western	1666	11.53
Mid-Eastern	1461	10.11
North-East	1389	9.61
South-Western	2367	16.38
West-Nile	1267	8.77
*Residence*		
Urban	2800	19.37
Rural	9307	80.41

^a^Age category missing 4 values

### Use of long-lasting insecticide-treated bed nets

Of the 14,450 participants, 11,884 reported to have slept under an LLIN the night before the survey, a percentage (95 % CI) of 80.10 (78.63–81.49). Among children under 5 years, 2511 children (83.12 %) out of 3021 slept under an LLIN. Households with more children under 5 years and women of child-bearing age (15–45 years) were significantly associated with using an LLIN (decreasing trends in odds of not using an LLIN) as shown in Table 2.

**Table 2:** Factors associated with not using a long-lasting insecticide-treated bed net

Variable	Status of LLIN usage number (%)		Crude OR (95 % CI)	Adjusted OR (95 % CI)^a^	p-value
	Yes N = 11,574	No N = 2876			
*Age categories* (*years*)					
0–5	2511 (83.12)	510 (16.88)	1	1	
6–14	2808 (75.26)	923 (24.71)	1.17 (1.38–1.90)	1.29 (1.11–1.49)	0.001^*^
15–45	4801 (80.86)	1136 (19.14)	1.17 (1.00–1.36)	1.02 (0.90–1.16)	0.745
>45	1450 (82.58)	306 (17.42)	1.04 (0.83–1.31)	0.69 (0.56–0.86)	0.001^*^
*Gender*					
Male	5542 (79.18)	1457 (20.82)			
Female	6033 (80.96)	1418 (19.04)	0.90 (0.81–0.99)	0.94 (0.85–1.04)	0.236
*Number of household members*					
1	354 (84.88)	62 (15.12)	1	1	
2	683 (81.43)	156 (18.57)	1.28 (0.87–1.89)	1.48 (0.97–2.24)	0.066
3	1235 (85.05)	217 (14.95)	0.99 (0.65–1.51)	1.34 (0.83–2.18)	0.228
4	1924 (80.76)	459 (19.24)	1.34 (0.94–1.90)	1.97 (1.27–3.07)	0.003^*^
5	1615 (79.53)	416 (20.41)	1.45 (1.01–2.06)	2.16 (1.38–3.38)	0.001^*^
6	2231 (79.59)	572 (20.41)	1.44 (0.98–2.12)	2.25 (1.36–3.71)	0.002^*^
>6	3532 (78.04)	994 (21.96)	1.58 (1.09–2.29)	2.27 (1.33–3.87)	0.003^*^
*Residence*					
Urban	2267 (80.98)	532 (19.02)			
Rural	9306 (79.88)	2344 (20.12)	1.07 (0.81–1.42)	1.06 (0.79–1.41)	0.695
*Number of sleeping rooms*					
1	3670 (82.21)	795 (17.79)	1	1	
2	3871 (80.65)	929 (19.35)	1.11 (0.91–1.35)	0.90 (0.72–1.12)	
3	2546 (78.45)	699 (21.55)	1.27 (1.02–1.57)	0.94 (0.73–1.21)	0.329
4	903 (77.68)	259 (22.32)	1.33 (0.99–1.79)	0.94 (0.67–1.32)	0.621
>4	421 (71.80)	165 (28.20)	1.81 (1.32–2.50)	1.25 (0.88–1.76)	0.732
*Spraying of rooms with insecticide*					
No	10,909 (80.06)	2717 (19.94)			
Yes	643 (80.29)	157 (19.71)	0.99 (0.65–1.49)	0.90 (0.88–1.76)	0.605
*Number of children under 5* *years*					
0	3433 (76.80)	1037 (23.20)	1	1	
1	3533 (81.37)	808 (18.63)	0.76 (0.63–0.92)	0.77 (0.61–0.98)	0.030^*^
2	3268 (81.12)	761 (18.88)	0.77 (0.63–0.94)	0.77 (0.61–0.96)	0.023^*^
>2	1340 (83.28)	269 (16.72)	0.66 (0.47–0.93)	0.67 (0.47–0.95)	0.027^*^
*Number of women of child bearing age*					
0	1784 (71.91)	697 (28.09)	1	1	
1	7213 (83.03)	1474 (16.97)	0.52 (0.44–0.62)	0.46 (0.38–0.57)	0.001^*^
>1	2576 (78.52)	705 (21.48)	0.70 (0.57–0.86)	0.49 (0.37–0.65)	0.001^*^
*Relationship structure*					
1 related adult	1784 (71.91)	331 (18.30)	1	1	
2 related adults	7213 (83.03)	1108 (17.25)	0.93 (0.73–1.18)	1.29 (0.96–1.1.74)	0.004^*^
3+ unrelated adults	2576 (78.52)	1437 (23.15)	1.34 (1.07–1.70)	1.60 (1.17–2.19)	0.870
*Community distribution of malaria medicine*					
No	6270 (81.15)	1456 (18.85)	1	1	
Yes	4840 (78.54)	1322 (21.46)	1.18 (0.99–1.39)	1.16 (0.97–1.38)	0.100
Do not know	452 (82.71)	95 (17.29)	0.90 (0.61–1.33)	0.87 (0.58–2.69)	0.504

^a^Adjusted for age, gender, number of household members, residence, number of sleeping rooms, spraying of rooms with insecticide, number of children under 5 years, number of women of child bearing age, relationship structure, community distribution of malaria medicine

* Statistically significant at p-value <0.05

The adjusted OR of not using an LLIN significantly decreased from the baseline in households that did not have a child under 5 years to those that had one or two children by 23 %, and further to 33 % if a household had more than two children. A similar trend of decreasing adjusted OR of not using an LLIN was observed among women of child bearing age, with a decrease of 54 and 51 % among households with one or more than one woman, respectively, compared to those that had no woman of child bearing age. However, the opposite was true for number of household members, the odds of not using an LLIN increased with increasing number of household members. Using households with one member as a baseline, there was significant increases in odds of not using an LLIN from households with five or more members. This ranged from 97 % in households with five members to 227 % in household with over six members. The same was true if a household had 3+ unrelated adults, with a 60 % increase in odds of not using an LLIN as compared to those that one related adult. Age categories gave a mixed picture, with children between 6 and 14 years having a 29 % likelihood of not using an LLIN compared to those below 5 years, however, individuals 45 years and older were more likely to use an LLIN. Other variables considered in the analysis, which were not significant and are not presented in the table included household wealth index and mother’s education level.

## Discussion

Eighty percent (80 %) of the population and eighty-three percent (83 %) of children under five years of age, in the households that achieved universal coverage, slept under an LLIN. This is a great accomplishment in this category considering that the WHO operation definition for success is the achievement of 80 % of the population that reports to have slept last night under an LLIN [1]. To put this achievement into perspective, this is an eleven point increase from the 69 % of all individuals’ surveyed and nine point increase from the reported 74 % among children under 5 years in the general population [13], an indication that universal LLIN coverage is a strong driver to achievement of optimal LLIN usage. The Ugandan NMCP adopted the strategy of universal coverage [6], and there are plans to carry out another mass distribution in the near future, therefore, understanding the usage behaviour among those that already have access to an LLIN is important, in line with this new direction.

These findings are dependable even if this analysis only included households that had achieved universal coverage, leaving out those that had also received LLINs in the mass campaign but did not fulfil this criterion (the reasons of which are beyond the scope of this study). This is so because the malaria indicator survey dataset used for this analysis had a very large sample size of a nationally representative unbiased population, even in the circumstances that only a subset (62 % of all households in the MIS) was used for this analysis, laying credence to the strength of our findings. However, because this was a cross sectional design, there was the likelihood of a biased positive response to having slept under an LLIN, especially soon after the mass distribution campaign where households had just received free LLINs.

One of the strengths of the mass distribution campaign is that it increases access and ultimately use of an LLIN, a result already observed in this analysis, especially among children under 5 years and women of child bearing age. However, two points of concern arose from this analysis, one directed to the behaviour change communication (BCC) and the second to the mass distribution campaign strategy. The first was that the use of LLINs among children between 6 and 14 years is significantly less when compared to those under 5 years possibly because of the current BCC messages that have mainly focused on their younger counterparts and pregnant mothers [20]. This is an important finding because the prevalence of malaria parasitaemia among children between the age of 6–14 years is quite high, especially in areas of high malaria transmission settings, like Uganda. They are a vital component for driving malaria transmission in these settings since they serve as reservoirs for malaria parasites [21, 22]. With the move towards achieving LLIN universal coverage, it is important to start packaging the message as ‘all inclusive’ since LLINs are bound to be accessed by the greater population, more so in light of the future planned mass distribution campaigns. The second was, as the number of household members increased, there was a significant increasing trend for not using an LLIN, in other words, the risk of not using an LLIN increased in households that had bigger numbers of household members. This could be explained with the notion that as the numbers of household members increase, not all of them are able to share an LLIN, leaving some of them to sleep without one. This is particularly significant during the planning phases of a mass distribution campaign exercise, which has so far implemented the ‘one size fits all’ strategy, for the number of LLINs given to a household (one LLIN for every two people). The consideration, in the future campaigns, to increase the number of LLINs to be given to households with more individuals, especially among those that have more than four numbers including covering all sleeping spaces [23], would be an additional option to the mass distribution campaign strategy.

## Conclusion

This analysis has showed that 80 % of the population used an LLIN among households that achieved universal coverage following the 2013 mass distribution campaign, especially among children under 5 years, an operational success. These findings can be generalized to the whole population, since the study sample was representative of the Ugandan population. This is encouraging especially in an era for the push to LLIN universal coverage, with strong evidence that access to an LLIN will lead to optimal LLIN usage. However, it is important to note that children between 6 and 14 years and individuals in household with more than four members were less likely to use an LLIN. In order to improve usage in these categories, it may require re-focusing the BCC message to be all inclusive especially at a time of increasing drive to achieve universal coverage, in addition to increasing the number of LLINs distributed in households with more than four household members in future mass distribution campaigns, respectively.

## Abbreviations

ANC: ante-natal clinic
BCC: behaviour change communication
DFID/UK: Department for International Development/United Kingdom
EA: enumeration areas
EPI: expanded programme for immunization
GFATM: global fund for AIDS, tuberculosis and malaria
LLIN: long-lasting insecticide-treated bed nets
MIS: malaria indicator survey
NMCP: National Malaria Control Programme
PMI: president’s malaria initiative
RBM: rollback Malaria
SBS-HDREC: Makerere University School of Biomedical Sciences Higher Degrees Research and Ethics Committee
UNCST: Uganda National Council for Science and Technology
UNICEF: United Nations Children’s Fund
VHT: village health team
WHO: World Health Organization
